# Metyrapone for Long-Term Medical Management of Cushing's Syndrome

**DOI:** 10.1155/2013/782068

**Published:** 2013-12-12

**Authors:** Andrea N. Traina, Ashley Farr, Ritu Malik, Robert J. Bingham

**Affiliations:** ^1^AstraZeneca LP, 1800 Concord Pike, Wilmington, DE 19803, USA; ^2^St. John Fisher College's Wegmans School of Pharmacy, 3690 East Avenue, Rochester, NY 14618, USA; ^3^Endocrine-Diabetes Care and Resource Center, Rochester General Health System, 224 Alexander Park, Suite 200, Rochester, NY 4607, USA; ^4^School of Medicine and Dentistry, University of Rochester, 601 Elmwood Avenue, Rochester, NY 14642, USA; ^5^Unity Diabetes Center, 2655 Ridgeway Avenue, Suite 220, Rochester, NY 14626, USA

## Abstract

Cushing's syndrome is characterized by any cause of excess cortisol in the blood and produces many physiologic consequences. Left untreated, Cushing's is associated with significant morbidity and mortality. Seventy percent of endogenous cases of Cushing's syndrome are secondary to a pituitary tumor; because of this, the primary mode of management is surgical resection of the tumor. Should hypercortisolism persist following surgical resection, further treatment options are limited. Metyrapone is an orphan medication that is often used in the diagnosis of the disease and occasionally for short-term treatment prior to surgery. Long-term treatment with metyrapone is usually discouraged due to the contradictory increase in ACTH production, acne, hirsutism, hyperkalemia, edema, and other mineralocorticoid effects. We present a patient with refractory Cushing's syndrome successfully treated for nearly 6 years with metyrapone with minimal adverse effects. This orphan medication may be a viable long-term treatment option for this difficult disease.

## 1. Introduction 

Cushing's syndrome is characterized by any cause of excess cortisol in the blood. Cortisol is a glucocorticoid that is responsible for the regulation of carbohydrate, protein, and lipid metabolism, it has anti-inflammatory properties, and its secretion is acutely increased during times of anxiety or stress. Cushing's syndrome causes many physiologic consequences including centripetal obesity, impaired immune response, generalized muscle weakness, menstrual irregularities, hypertension, and premature death secondary to cardiovascular disease, infection, or suicide [1, 2]. Iatrogenic Cushing's syndrome caused by exogenous glucocorticoid administration is the most common cause of excess cortisol levels. Of patients with endogenous causes of Cushing's syndrome, 70% are secondary to a pituitary tumor; because of this, the primary mode of management is surgery to remove the tumor. Transsphenoidal surgery has up to an 80% remission rate for microadenomas and 50% for macroadenoma removal [3–5]. Should hypercortisolism persist following surgical resection, further treatment options are limited; a second attempt at transsphenoidal surgery, pituitary irradiation, adrenalectomy, steroidogenic inhibitors, or a combination of strategies is all that remains.

The available steroidogenic inhibitors are difficult to use secondary to poor tolerability and lack of data regarding long-term efficacy. As a result, pharmacological treatment is often reserved for refractory Cushing's, failed surgical cases, or patients who refuse or are not surgical candidates [3, 6, 7]. Metyrapone is an orphan medication that holds the FDA approval for the diagnosis of Cushing's syndrome and is occasionally used off-label for short-term treatment of Cushing's syndrome prior to surgery. Metyrapone prevents cortisol synthesis by inhibiting 11 *β*-hydroxylase, the enzyme responsible for the conversion of deoxycortisol to cortisol. Metyrapone's restricted access and adverse reactions associated with increased androgen and mineralocorticoid production limit the use of this medication for the treatment of Cushing's syndrome [6, 7]. We report the off-label use of metyrapone as a successful long-term management of refractory Cushing's syndrome. Our patient demonstrated significant improvement in her symptomatology, laboratory parameters, and quality of life with metyrapone use after two failed attempts at a surgical cure.

## 2. Case Presentation 

A 44-year-old Caucasian female was diagnosed with Cushing's syndrome after referral to our endocrinology office for amenorrhea for 4 years and osteoporosis diagnosed by DXA scan. Prior to the referral, her past medical history included hyperlipidemia, hypertension, depression, and congenital deafness. Per patient interview, she reported increased fatigue, emotional fluctuations, muscle weakness, and ease of bruising over the last several years. She also explained a distant history of amenorrhea and a pituitary tumor 20 years prior for which she took medication that made her nauseous that she subsequently discontinued after approximately 4 months of treatment. On physical exam, she presented with a blood pressure of 120/82 mm Hg and a resting pulse of 76; she displayed slight facial plethora, a tremor of the outstretched hands, and slightly brisk reflexes. There was no evidence of galactorrhea and the remainder of her physical exam was unremarkable. Following this initial visit, laboratory tests were ordered revealing mostly normal results, including a prolactin of 26 ng/mL (3–29 ng/mL). Her only abnormal result was an elevated AM cortisol of 26.3 mcg/dL (5–23 mcg/dL). A 1 mg overnight dexamethasone suppression test revealed an AM cortisol of 20.4 mcg/dL (<5 mcg/dL). An abnormal 2-day low dose dexamethasone suppression test as well as a 24-hour urine-free cortisol of 194 mcg/24 hr (20–90 mcg/24 hr) established the presence of Cushing's syndrome.

In March of 2005, an MRI revealed a 4 mm microadenoma in the left aspect of the pituitary gland. An inappropriately elevated serum adrenocorticotropin hormone (ACTH) level further supported the diagnosis of Cushing's syndrome. A corticotropin-releasing hormone (CRH-) induced inferior petrosal sinus sampling (IPSS) was ordered and attempted; however, after unsuccessful cannulation of the left internal jugular vein, the results were unreliable. The decision was made to continue with surgical removal of the tumor and in August of 2005, the patient underwent transseptal sublabial transsphenoidal surgery to remove her pituitary tumor. Surgical pathology revealed an ACTH negative pituitary microadenoma, inconsistent with her clinical history of Cushing's syndrome.

Despite the surgical pathology, her AM cortisol, 24-hour urine-free cortisol level, and Cushing's symptoms began improving soon after surgery. In November 2005, she noted improved mood, decreased muscle weakness, and resumption of her menses and her 24-hour urine-free cortisol was 101 mcg/24 hr. Unfortunately, in February of 2006, her 24-hour urine-free cortisol level was again elevated at 413 mcg/24 hr. A repeat MRI indicated persistent evidence of a left sided pituitary adenoma that was likely responsible for the patient's persistent Cushing's syndrome.

The patient was again referred to neurosurgery for evaluation and the recommendation was made for repeat transsphenoidal surgical resection. In October of 2006, surgery was performed and pathological examination confirmed the removal of an ACTH positive pituitary tumor. In November of 2006, the patient again reported improved mood but increased fatigue and generalized myalgias, likely due to the reduced serum cortisol levels. Postoperatively, the patient was receiving prednisone 5 mg daily to prevent secondary hypoadrenalism. In December of 2006, she reported improved energy, normal menses, lessened anxiety, and weight loss. In January of 2007, her AM cortisol level was 20.3 mcg/dL without her 5 mg of prednisone daily. Her exogenous glucocorticoid supplementation was discontinued at this time and in February, her 24-hour urine-free cortisol was 99 mcg/24 hr.

After months of symptom improvement, the patient presented for a follow-up visit with neurosurgery in September 2007, with a 24-hour urine-free cortisol of 165 mcg/24 hr. At this time, she was presented with three options: repeat pituitary surgery via complete hypophysectomy, ketoconazole with pituitary radiation, or bilateral adrenalectomy with pituitary radiation. She returned to our endocrinology office to discuss her options; after two failed surgeries she was hesitant to agree to a third. At the recommendation of our endocrinologist, she agreed to compassionate use of metyrapone in combination with pituitary radiation. The patient was initiated on metyrapone 250 mg once daily and this was titrated up to 250 mg twice daily after 1 week. One month following metyrapone initiation, her AM cortisol level was normal at 17.5 mcg/dL and her 24-hour urine-free cortisol was normal at 81 mcg/24 hr. The patient tolerated metyrapone therapy extremely well with no significant adverse reactions. She noted continued regular menses, improvement in mood, energy, and decreased myalgias following initiation of metyrapone. Every three months while on therapy, we obtained an electrolyte panel, 24-hour urine-free cortisol, AM cortisol, prolactin level, thyroid stimulating hormone, free thyroxine, and an IGF-1 level to monitor for changes in pituitary function as the pituitary radiation began taking effect.

In July 2009, at her follow-up appointment, the patient presented with a blood pressure of 153/91 mm Hg (repeat 142/100 mm Hg), a pulse of 98, fatigue, occasional dizziness, and weight gain. Her 24-hour urine-free cortisol level was 150 mcg/24 hr at this point and her metyrapone dose was increased to 250 mg three times daily. One month later her complaints had resolved, her blood pressure was 127/83 mm Hg, and her 24-hour urine-free cortisol was 81 mcg/24 hr. Following this dose adjustment, she has continued to tolerate the metyrapone without difficulty, has remained symptom free, and maintained 24-hour urinary cortisol levels within the normal range. Her most recent pituitary MRI showed no significant changes from the previous study in 2008.

Metyrapone is only available from the manufacturer on a compassionate use basis; every six weeks, Novartis pharmaceuticals is contacted requesting a 2-month supply of metyrapone be shipped to our office for the patient to pick up. At the time of writing, the patient has been stable on metyrapone for 70 months of therapy; she has tolerated it well with consistently normal laboratory parameters.

## 3. Discussion 

Long-term treatment with metyrapone is usually discouraged due to the contradictory increase in ACTH production triggered by the negative feedback loop, acne, hirsutism, hyperkalemia, edema, and other mineralocorticoid effects. Metyrapone has historically been successful in treating Cushing's patients on a short-term basis and the side effects have shown to be tolerated in this setting [6, 7].

Pituitary radiation can take several years to take its full effect and the complications of remaining cushingoid until that point are associated with significant morbidity and mortality [1–3]. Ketoconazole is often used as an adjunct therapy; however, it is often poorly tolerated and metyrapone may be a more favorable option. The endocrinologist treating this patient had used metyrapone in a similar case of recurrent Cushing's with much success. Metyrapone was well tolerated in that patient and their cortisol levels were controlled until radiation therapy had taken its full effect.

Traditionally, pharmacological treatment options for Cushing's syndrome consist of ketoconazole, mitotane, and metyrapone [3, 6, 7]. There are two newer agents available for the treatment of Cushing's syndrome: pasireotide and mifepristone; neither was available when this patient required initial pharmacologic intervention and given her excellent response to metyrapone their availability did not change our treatment course [8, 9].

Ketoconazole blocks steroidogenic cytochrome P450 enzymes which lowers plasma cortisol but requires an acidic environment and can cause gastrointestinal adverse reactions, hypogonadism in male patients, and abnormal liver function tests [7]. Mitotane is only used for treatment of cortisol-producing adrenal carcinomas as it is taken up by both healthy and malignant tissue and causes a wide array of toxicities [6]. Pasireotide treatment is successful in up to 25% of patients and is a somatostatin analog administered twice daily as a subcutaneous injection. It has high incidences of hyperglycemia (40%), cholelithiasis (30%), headache, and gastrointestinal distress [8]. Mifepristone is a cortisol receptor blocker and is used mainly to decrease the hyperglycemia induced by hypercortisolism in Cushing's patients. The long-term use for Cushing's is unknown and it has a high incidence of gastrointestinal and central nervous system adverse reactions [9]. As previously discussed, metyrapone inhibits 11 *β*-hydroxylase, the enzyme that converts 11 deoxycortisol into cortisol. The patient in this case tolerated metyrapone extremely well and showed significant biochemical and symptomatic improvement.

## 4. Conclusions

Surgery is the first line for the treatment of Cushing's disease and should remain the mainstay of management. Few medications have been proven to be beneficial for patients who cannot undergo surgery or when surgery is unsuccessful. Although metyrapone is not FDA approved for the long-term treatment of Cushing's disease, in conjunction with radiation therapy, it has been shown to be valuable in the treatment of Cushing's by suppressing endogenous cortisol production. The reported side effects of metyrapone have been minimal in this patient's case. Although there are newer agents available for the treatment of Cushing's syndrome, long-term treatment with metyrapone should remain a viable treatment option.
